# The influence factors of medical professionalism: A stratified-random sampling study based on the physicians and patients in ambulatory care clinics of Chengdu, China

**DOI:** 10.1097/MD.0000000000005128

**Published:** 2016-10-28

**Authors:** Yifei Lin, Senlin Yin, Sike Lai, Ji Tang, Jin Huang, Liang Du

**Affiliations:** a Department of Urology, Institute of Urology (Laboratory of Reconstructive Urology), West China Hospital, Sichuan University, Chengdu, Sichuan, P.R. China; b Department of Neurosurgery, West China Hospital, Sichuan University, Chengdu, Sichuan, P.R. China; c West China School of Medicine, Sichuan University, Chengdu, Sichuan, PR China; d Global Services, NewYork-Presbyterian Hospital, New York, NY, U.S.A; e West China Hospital, Sichuan University, Chengdu, Sichuan, P.R. China.

**Keywords:** medical professionalism, payment, satisfaction

## Abstract

As the relationship between physicians and patients deteriorated in China recently, medical conflicts occurred more frequently now. Physicians, to a certain extent, also take some responsibilities. Awareness of medical professionalism and its influence factors can be helpful to take targeted measures and alleviate the contradiction. Through a combination of physicians’ self-assessment and patients’ assessment in ambulatory care clinics in Chengdu, this research aims to evaluate the importance of medical professionalism in hospitals and explore the influence factors, hoping to provide decision-making references to improve this grim situation. From February to March, 2013, a cross-sectional study was conducted in 2 tier 3 hospitals, 5 tier 2 hospitals, and 10 community hospitals through a stratified-random sampling method on physicians and patients, at a ratio of 1/5. Questionnaires are adopted from a pilot study. A total of 382 physicians and 1910 patients were matched and surveyed. Regarding the medical professionalism, the scores of the self-assessment for physicians were 85.18 ± 7.267 out of 100 and the scores of patient-assessment were 57.66 ± 7.043 out of 70. The influence factors of self-assessment were physicians’ working years (*P* = 0.003) and patients’ complaints (*P* = 0.006), whereas the influence factors of patient-assessment were patients’ ages (*P* = 0.001) and their physicians’ working years (*P* < 0.01) and satisfaction on the payment mode (*P* = 0.006). Higher self-assessment on the medical professionalism was in accordance with physicians of more working years and no complaint history. Higher patient-assessment was in line with elder patients, the physicians’ more working years, and higher satisfaction on the payment mode. Elder patients, encountering with physicians who worked more years in health care services or with higher satisfaction on the payment mode, contribute to higher scores in patient assessment part. The government should strengthen the medical professionalism for young physicians and improve the payment mode.

## Abstract 2

Supplemental Digital Content is available in the text

## 1 Introduction

Medical professionalism was first proposed in “Medical Professionalism in the New Millennium: A Physician Charter” issued by the American Internal Medicine Foundation, ACP (American College of Physicians) Foundation and the European Federation of Internal Medicine in 2002.^[1]^ One hundred and twenty international medical organizations in >30 countries or regions have signed the declaration so far. Construction Committee of the Chinese Medical Association Ethics also issued the “Chinese Physician Charter” in 2005. These documents reveal the importance of professionalism in health care services. Meanwhile, medical professionalism is the premise and guarantee of positive physician–patient communication. The experience of the world-famous Mayo Clinic has shown that medical professionalism was the core competitiveness of medical institutions.^[2]^ Therefore, medical professionalism can be regard as the backbone of physicians and even of the medical industry.

Nowadays in China, physician–patient relationship becomes worse than before, even with the advancement of medical reform, forcing the public and the government to consider the reasons for this dilemma.^[^3–10^]^ Since the relationship can be influenced by the medical professionalism profoundly to a certain extent, it is of great significance for the medical field, even the whole society, to explore the influence factors so as to foster the relationship. Nonetheless, current relevant studies, most of which were based on the foreign status exempted China, are theoretical analysis with less solid evidence or informative data. Quite a few studies related to China were retrieved and western regions are especially a blank.

Western area of China is densely populated, but with relatively week health care force. The contradiction between physicians and patients becomes extremely prominent in this region. Thus, it is more meaningful to study and discuss on the medical professionalism in this region. Through a combination of physicians’ self-assessment and patient-assessment, this research aims to evaluate the importance of medical professionalism and explore the influence factors in ambulatory care clinics from different class and different system in Chengdu, hoping to provide decision-making references to improve medical professionalism and future medical reform.

## 2 Methods

### 2.1 Sample selection

From February to March 2013, a cross-sectional study was conducted in Chengdu city, China. We used a stratified sampling to randomly select 2 tier 3 hospitals, 5 tier 2 hospitals, and 10 community hospitals. Classified by departments, we randomly picked the open clinics on the same day and conducted the survey to the physicians as well as his/her former 5 admissions of patients. When there were refusals to our survey, patients sequent visited would be included until we had 5 of them.

### 2.2 Survey design and data collection

A combination of physicians’ self-assessment and patient-assessment was conducted. Questionnaires are adopted version based on the surveys designed by Chisholm et al^[11]^ and Mackillop et al.^[12]^ Both surveys consist of basic characteristic part and medical professionalism part. Physicians’ basic characteristic incorporates professional ranks, educational background, age, hospital class, payment strategy, physician income, and so on. Patients’ basic characteristic included: age, gender, education, and purpose of this visit.

In terms of the medical professionalism part, Marie proposed a physician self-assessment survey that is evaluated from 6 dimensions, including altruism, accountability, excellence, duty, honor and integrity, respect for others. Twenty questions of self-assessment with a total of 100 scores were assessed and each ranged from 1 to 5 scores. And Mackillop et al designed a patient-assessment form according to a recent public survey, carried out for the GMC (General Medical Council), which identified the qualities of a physician that were most important to patients were: giving good advice and treatment, communication skills, maintaining confidentiality, respecting dignity, and being involved in treatment decisions. Fourteen questions of patient-assessment with a total of 70 scores were marked and each also ranged from 1 to 5 scores.

Pilot survey was applied in order to modify and polish the questionnaires, which guarantees the smooth progress of the final survey, as well as objective and accurate results. Additionally, experts from Center for the Study of Society and Medicine, Center on Medicine as a Profession of Columbia University also helped us revise the questionnaires.

The survey required face-to-face interview and all the interviewers were trained before the practice. Doctors themselves filled the physicians’ surveys, whereas the patients’ were by the interviewers so as to ensure that questionnaires were completed accurately. The data collected from the patients were anonymized prior to analysis. Before the participants filled in the form, our trained interviewers emphasized the informed consent orally. Thus, the ethic statement was not necessary for the research. Informed consent was written on the top of the questionnaire and asked the interviewer to read it carefully.

### 2.3 Statistical analysis

Data were entered in EpiData 3.1 and analyzed using SPSS version 17.0. Categorical data was described by frequency and constituent ratio, whereas measurement data was described using mean ± standard deviation. Regarding the influence factors of physician professionalism, we first clustered all the characteristics of physicians and patients using cluster analysis. After excluding the interactive variables, forward regression analysis was adopted for selecting the significant influence factors of physician professionalism.

Factors analysis of physician professionalism includes 3 parts, that is, the impact of physicians’ baseline characters on the self-assessment of physician professionalism, the impact of physicians’ baseline on the patients’ evaluation of physician professionalism, and the impact of patient's baseline on the patients’ evaluation of physician professionalism.

## 3 Results

### 3.1 Baseline characteristics

**Figure 1:**
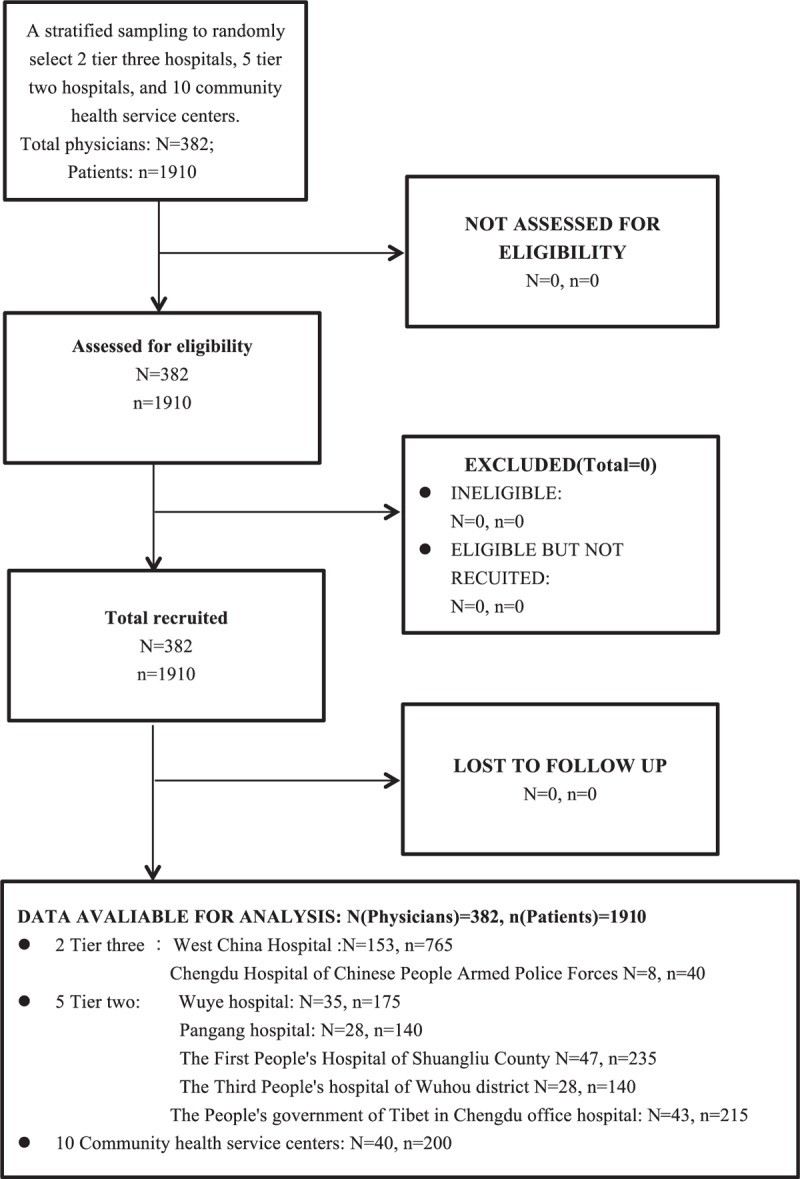
The STROBE flowchart.

The baseline characteristics of the physicians and patients were listed in Table 1 . We investigated 382 physicians from 2 tier 3 hospitals (West China Hospital and Chengdu Hospital of Chinese People Armed Police Forces), 5 tier 2 hospitals (The People's government of Tibet in Chengdu Office Hospital, The Third People's hospital of Wuhou district, Wuye Hospital, Pangang Hospital, The First People's Hospital of Shuangliu County), and 10 community hospitals. The STROBE flowchart was in Fig. 1.

**Table 1:**
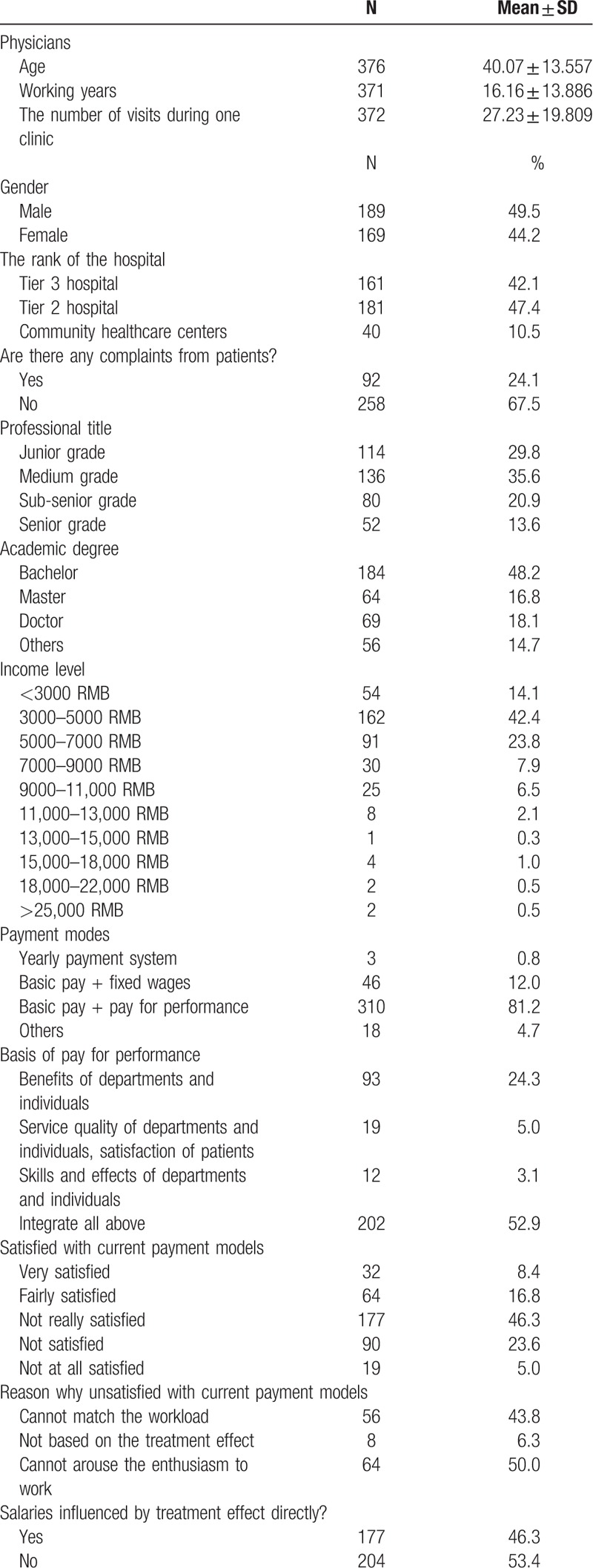
Baseline characteristic of the physicians and patients.

**Table 1 (Continued):**
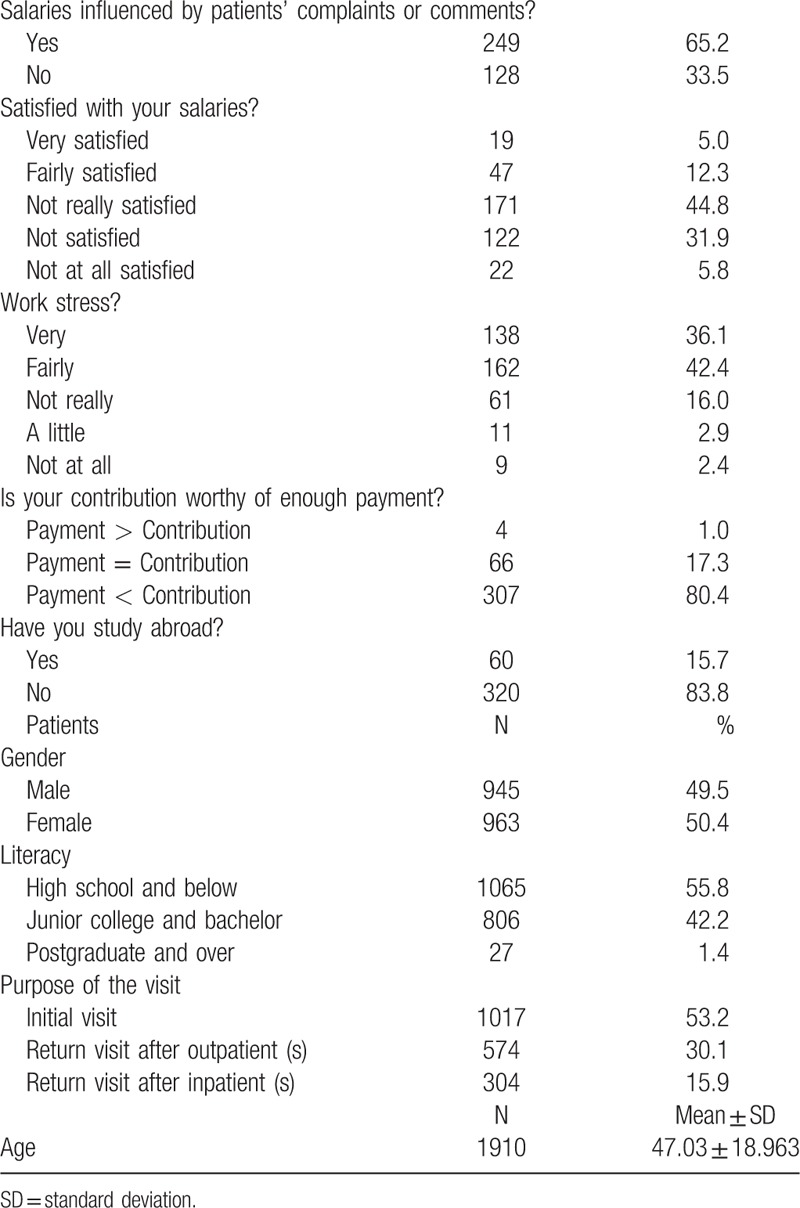
Baseline characteristic of the physicians and patients.

A total of 1910 patients (average age 47.03 ± 18.96 years old, median age 47 years old) were recruited. The percentage of male patients was 46.9%. In terms of educational background, patients who have attended middle school or below consisted of 55.8% of the patients, whereas patients with postgraduate or higher degree consisted only 1.4%. Among the interviewed patients, 53.2% were first-visit patients, whereas returning outpatient visit patients and discharged inpatient return visit patients accounted for 30.1% and 15.9% respectively.

### 3.2 Evaluation of medical professionalism

#### 3.2.1 Self-assessment of medical professionalism

The results of self-assessment of medical professionalism were presented in Table 2. The average overall score of self-evaluation of medical professionalism was 85.18 ± 7.26 (median 85.5), and average scores of 6 domains of the respect for others, honor and integrity, excellence, duty, accountability, altruism and humanitarian were 17.09 ± 2.08, 11.27 ± 2.01, 13.34 ± 1.53, 8.93 ± 0.97, 13.76 ± 1.40, and 20.79 ± 2.81, respectively.

**Table 2:**
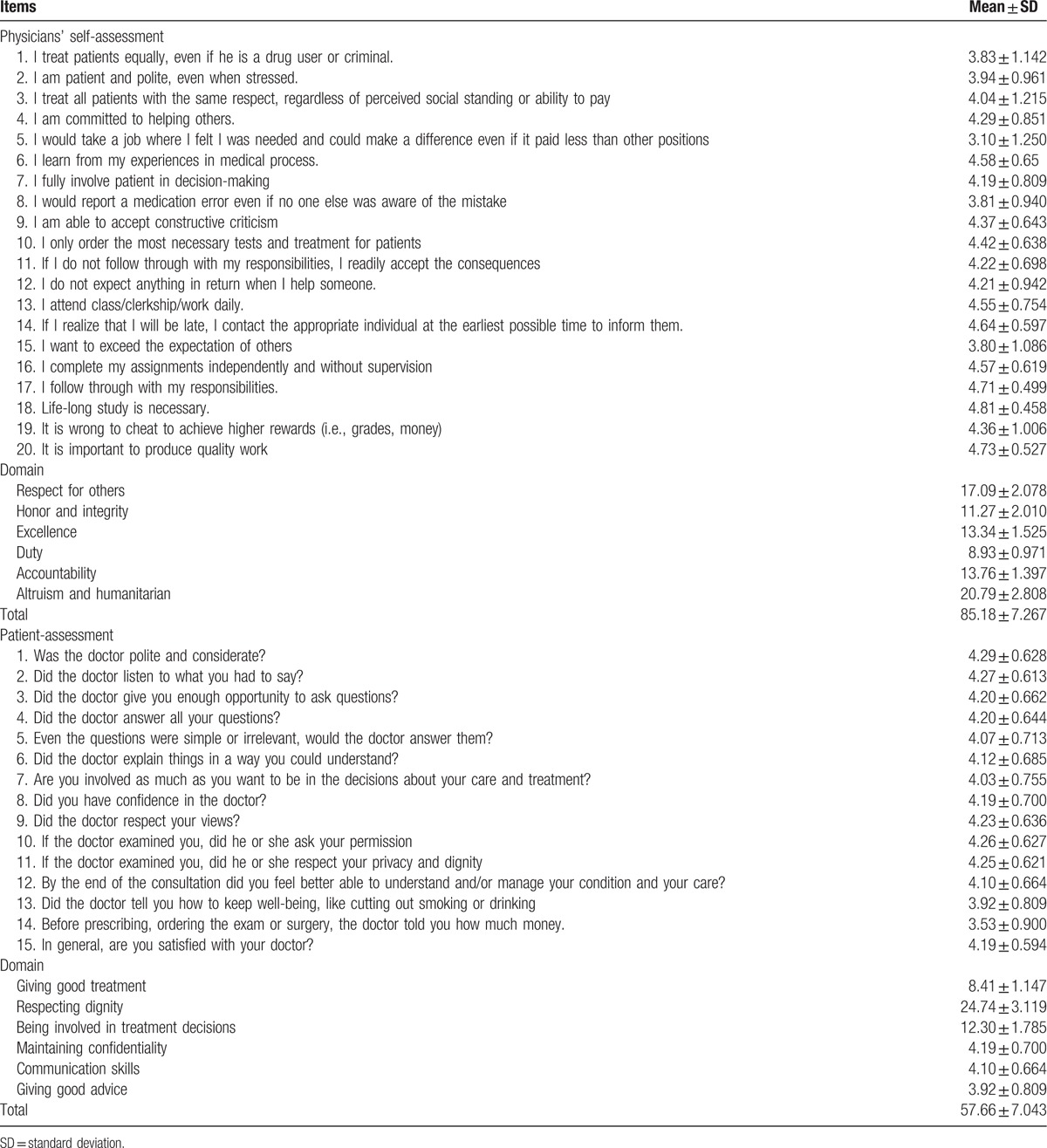
The results of physicians’ self-assessment and patient-assessment of medical professionalism.

#### 3.2.2 Patient evaluation of medical professionalism of physicians

The results of patient evaluation of medical professionalism were listed in Table 2. The overall score was 57.66 ± 7.04 (median score 54.00). The scores of 6 domains of giving good treatment, respecting dignity, being involved in treatment decisions, maintaining confidentiality, communication skills, giving good advice were 8.41 ± 1.15, 24.74 ± 3.12, 12.30 ± 1.79, 4.19 ± 0.70, 4.10 ± 0.66, and 3.92 ± 0.81, respectively.

#### 3.2.3 Cluster analysis of influential factors on medical professionalism

The results of cluster analysis of influence factors on medical professionalism were presented in Appendix 1 and 2. The influential factors from patients were clustered into 2 categories (Literacy of patients and Purpose of the visit), whereas the influential factors from physicians were clustered into 4 categories (“Are there any complaints from patients?” “Working years.” “Age of physicians,” and “The number of visits during one clinic”).

#### 3.2.4 Forward regression analysis

Based on the cluster analysis of baseline characteristics of physicians and patients, we performed forward regression and found that the influential factors on self-evaluated medical professionalism were long working years (*P* = 0.003) and no history of complaint (*P* = 0.006), which was presented in Table 3. In particular, self-evaluation of the domain of Duty (*P* = 0.025) and accountability (*P* < 0.01) was associated with only the working years. The honor and integrity domain was only associated with history of complaints (*P* = 0.006). Meanwhile, the domains of respect for others and altruism/humanitarian were associated with both factors.

**Table 3:**
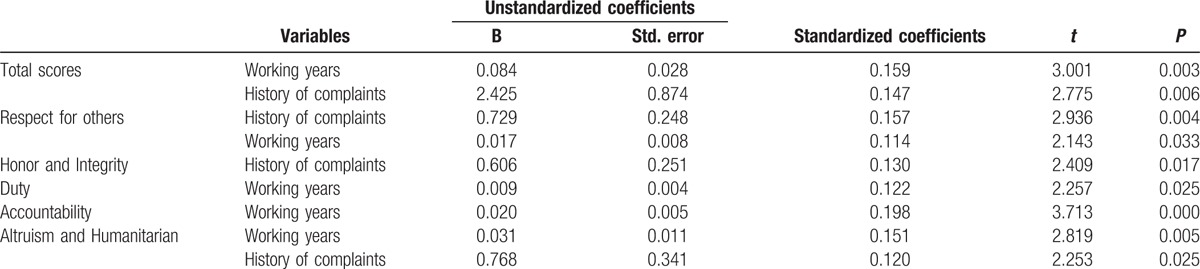
The impact of physicians’ baseline characteristics on the self-assessment of physician professionalism.

The influence factors on patient-evaluation of medical professionalism of physicians were patients’ age (*P* = 0.001), working years of physician (*P* < 0.01), and the satisfaction level of physician toward the payment models (*P* = 0.006), which were presented in Table 4. In particular, among the baseline characteristics of patients, the domains of giving good treatment (*P* < 0.01), being involved in treatment decision (*P* = 0.001), and giving good advices (*P* = 0.007) were associated with patients’ age. Meanwhile, the gender of patients was also one of the influential factors in terms of domains of respecting dignity (*P* = 0.048) and maintaining confidentiality (*P* = 0.015).

**Table 4:**
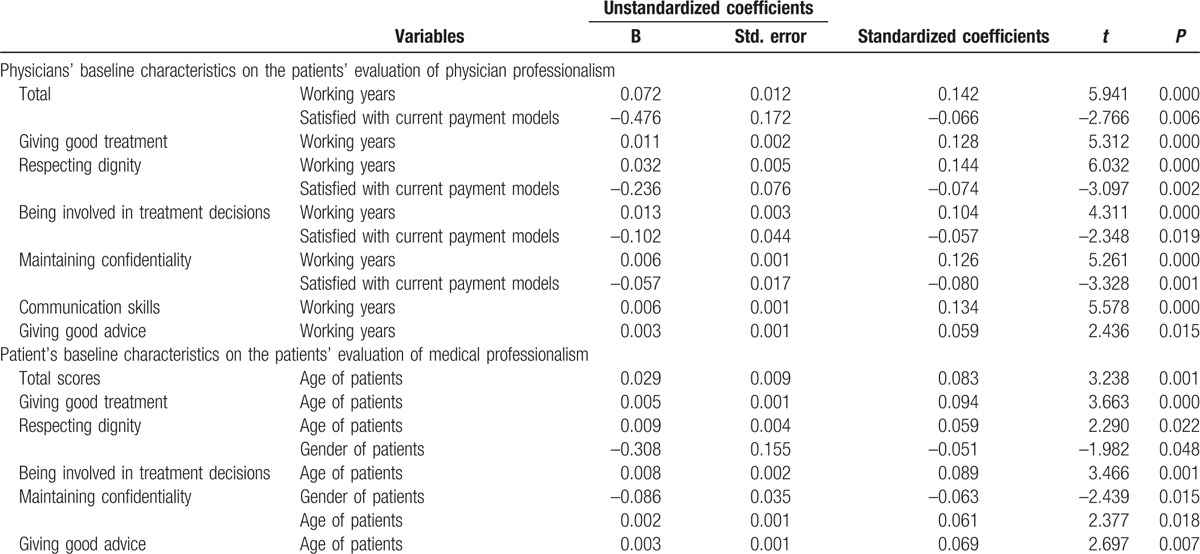
The impact of physicians’ baseline and patient's baseline characteristics on the patients’ evaluation of physician professionalism.

As for the baseline characteristics of physicians, the domains related only to the working years of the physicians were giving good treatment (*P* < 0.01), communicating skills (*P* < 0.01), and giving good advice (*P* = 0.015). The scores of other domains were simultaneously influenced by the practicing years and satisfaction level toward the payment models, for instances: respecting dignity (*P* < 0.01, *P* = 0.002), being involved in treatment decision (*P* < 0.01, *P* = 0.019), and maintaining confidentiality (*P* < 0.01, *P* = 0.001)

## 4 Discussion

Patients were generally satisfied with their physicians. The mean scores of physicians’ self-assessment and the patients’ evaluation on medical professionalism both represent positive feedback. According to the statistical analysis, higher scores of self-assessment on the medical professionalism were in accordance with physicians’ more working years and no complaints history. Elder patients tend to mark higher scores on their physicians. Physicians with more working years and higher satisfaction level on the payment modes got higher scores from patients.

First and foremost, physicians’ working year is the fundamental factor that affects the medical professionalism. For one thing, as working year reflects experience of physicians to some extent, physicians, after decades of practice, can be more expert and proficient. In the face of patients, the veteran physicians are more confident and flexible to apply the theory and practical skills, then better coping with the physician–patient relationship. Thus, higher self-confidence for clinical decisions and behavior leads to better self-assessment. For another, patients, so as most people, tend to judge others by appearance. Elder physicians look like more skillful. It is long practice time that makes them accumulate work experience, such as communication skills.^[^13–15^]^ Then, a more persuasive and convinced physician will have closer follow-up of patients and even better treatment outcomes. In turn, patients marked higher scores on the physicians accordingly.

In addition, history of patients’ complaints can also affect the medical professionalism. It is obviously delightful to work when physicians got no complaints from patients. They will think highly of their own job and have higher self-assessment scores. However, more than two-thirds of physicians claimed that patients’ complaints and comments could impact their incomes. When at risks to be complained, physicians may consider more about not losing money rather than how to provide appropriate treatment and advice. As a result, the self-assessment of Respect for others, Altruism/Humanitarian and Honor/Integrity turn to be lower. Van Mook^[15]^ from a Holland teaching hospital investigated 140 complaints in 5 years and found that the complaints focus more on medical professionalism instead of medical errors. The external influence factor has a mutual effect between the patients’ complaints and medical professionalism, which need the public's attention.

Furthermore, the satisfaction level towards the payment mode is another essential factor. In the sight of hospital leadership, mode of payment is the core of human resource management and is essential for attracting and retaining talents; in the sight of doctors, mode of payment has the functions of security and motivation. Nevertheless, this study presents a grim situation that near four-fifths of physicians surveyed were not really satisfied with their payment mode. More than half of physicians surveyed complained that it was not incentive enough. Meanwhile, the satisfaction level towards the payment mode also reflects the cognition of physicians on workload and salaries. From the baseline characteristic of physicians, two-thirds of physicians complained about the work stress and 80% argued that payment was less than the value of contribution. Hence, unsatisfied payment modes resulted in physicians’ lack of motivation. Patients then felt uncomfortable with the indifferent physician and marked low medical professionalism scores in response.

Under no circumstances can we ignore the age issue. In current China, particularly in western region, elder patients, most of whom got lower degree of education and were hard to get access to up-to-date information, preferred to believe in authority thus having a close relationship with physicians.

By contrast, younger patients were more critical; in addition, they have high expectations of the treatment outcomes. As the young have more access to information, they may be misled by some non-objective news, and then they become doubtful about the clinical decisions from their physicians. Therefore, the poor follow-ups led to the undervaluation of their physicians. Results from Leung’ s research^[16]^ also found similar phenomenon in Queen Mary Hospital, 1 of the only 2 teaching hospitals in Hong Kong. Interestingly, Tanco et al^[17]^ also claimed that trust was associated with older age patients. Patients trust in physicians is essential for a smoothly-operated healthcare system,^[18]^ but the far-reaching and rapid transformation of the health care system have resulted in great pressure on patient–physician trust and may even be undermining it. Therefore, it is of significance to improve patients trust to physicians.^[^19 20^]^

We initially supposed that payment mode and physicians’ salaries would play a significant role on medical professionalism, which is confirmed by some studies.^[^21 22^]^ Yet this study concluded a negative outcome. Possible reasons may be the inconsistent payment incentive standards in different hospitals and the difference in awareness of physicians.

Although this study did not find medical professionalism influenced by payment modes, most researches from the United Kingdom or other countries believed that the payment modes played a crucial role. Foreign physician payment mode is currently conversing from “fee-for-service” mode to “pay-for-performance” mode^[23]^; so most of the researches explore the effect of “pay-for-service” mode on medical professionalism. Qaseem and others^[21]^ explore in 4 dimensions (the application of scientific evidence, ethics of interaction between physicians and patients, the pursuit of fairness and commitment to professionalism), pointing out that “pay-for-service” mode can stabilize medical professionalism. However if it is poorly designed, adverse effects would come out; good “pay-for-service” mode enables physicians find balance between external motivation and self-interest, intrinsic motivation and self-restraint.

Moreover, whether or not the physicians got medical professionalism training,^[^24–30^]^ whether or not the training was scientifically designed^[^31 32^]^ were also relevant. Thus, the domestic situation asks for more exploration.

There are several limitations to our study. First, some physicians filled the questionnaires absently due to the crowded clinics and nurses or other medical staff could possibly affect the procedure of assessments. In addition, our trained interviewers guaranteed that the physicians would not see the results and all the surveys were anonymous. However, several numbers of patients still doubted it and were afraid of being treated improperly by their physicians if negative comments were made. Third, though the questionnaires were refined and polished by the experts of relevant field, and revised thanks to the results of the pre-survey, some conditions were ignored, for instance, the limitations of region suggest cautious application and interpretation of the results.

In spite of the limitations, our study is of significance owing to the fact that domestic researches in this field can hardly be found. Our research is the first one that focuses on western area of China. Our results found that patients were generally satisfied with their physicians and the influence factors on medical professionalism need to be taken seriously. The government needs to strengthen the medical professionalism for young physicians. Stronger emphasis in future surveys should be paid to measuring the whole Chinese medical professionalism status.

## 5 Conclusion

Higher self-assessment on the medical professionalism was in accordance with physicians of more working years and no complaint history. Higher patient-assessment was in line with elder patients, the physicians’ more working years and higher satisfaction on the payment mode. Elder patients, encountering with physicians who worked more years in health care services or with higher satisfaction on the payment mode, contribute to higher scores in patient assessment part. The government should strengthen the medical professionalism for young physicians and improve the payment mode.

## Acknowledgments

The authors thank Prof. David J. Rothman and Prof. Sheila Rothman from Center for the Study of Society and Medicine, Center on Medicine as a Profession of Columbia University, and Prof. Yali Cong from Department of Medical Humanities, Peking University Health Science Center for their help for design of methods and modification of the questionnaires.

## Supplementary Material

Supplemental Digital Content
