# Supplementary material for: The influence factors of medical professionalism: A stratified-random sampling study based on the physicians and patients in ambulatory care clinics of Chengdu, China
Source: Medicine (Baltimore). 2016 Oct 28;95(43):e5128. doi: 10.1097/MD.0000000000005128 (PMC5089095; doi:10.1097/MD.0000000000005128)
Supplement: Supplemental Digital Content [file medi-95-e5128-s001.doc]

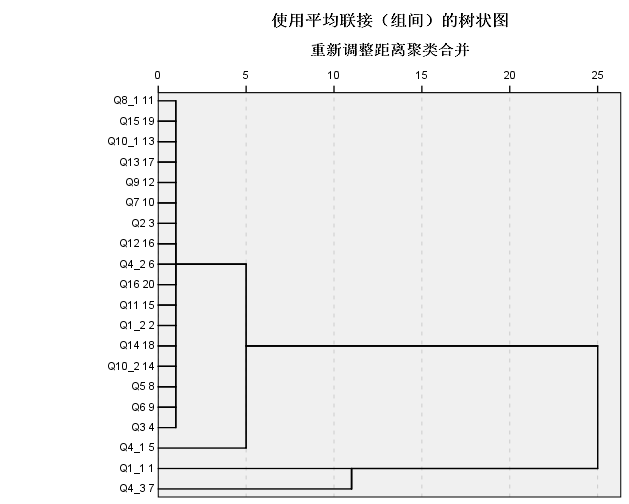


**Appendix 1. Clustering analysis of influence factors from the physicians**

Note: Using the average connection between groups of tree to adjust the distance clustering merge

Q1-1 1: Age of physicians

Q1-2 2: Gender of physicians

Q2 3: The nature of the hospital(all are public hospitals.)

Q3 4: The rank of the hospital

Q4-1 5: Working years

Q4-2 6: Is there any complaints from patients?

Q4-3 7: The number of visits during one clinic

Q5 8: Professional title

Q6 9: Academic degree

Q7 10 Income level

Q8-1 11 Payment modes

Q9 12: Basis of pay for performance

Q10-1 13: Satisfied with current payment models

Q10-2 14: Reason why unsatisfied with current payment models

Q11 15: Salaries influenced by treatment effect directly?

Q12 16: Salaries influenced by patients' complaints or comments?

Q13 17: Satisfied with your salaries?

Q14 18: Work stress?

Q15 19: Is your contribution worthy of enough payment？

Q16 20: Have you study abroad?


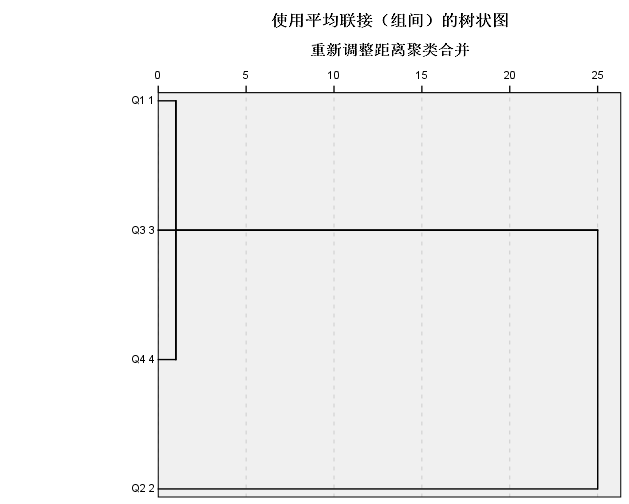


**Appendix 2. Clustering analysis of influence factors from the patients**

Note: Using the average connection between groups of tree to adjust the distance clustering merge

Q1 1: Age of patients

Q2 2: Gender of patients

Q3 3: Literacy of patients

Q4 4: Purpose of the visit
